# The National Insurance Act (Part II.) Amendment Bill

**Published:** 1914-05-09

**Authors:** 


### NATIONAL INSURANCE ACT DEVELOPMENTS.

### The National Insurance Act (Part II. Amendment) Bill.

A Bill has recently been introduced into the House of Commons having for its object the amendment of certain of the provisions of the Act of 1911 dealing with unemployment insurance. Although the subject of unemployment insurance is at present of practical interest only to a somewhat limited section of the community, yet in view of the probability of considerable extension in its scope in the future it is important that any developments which take place should be carefully observed and the general trend of any alteration of existing machinery considered. We therefore make no apology for dealing this week with a Bill which, if passed into law, will effect certain amendments of Part II. of the Act of 1911.

### Future of Unemployment Insurance.

As indicated in a Memorandum by which the Bill is prefaced, no considerable changes of policy are involved; the general direction of the Bill is to remove certain administrative difficulties experienced by the Board of Trade, by employers, and by workmen. In this connection it may be remarked that the administration of the unemployment provisions of the Act of 1911 does not appear to have been attended with anything like the difficulty and dissatisfaction which have accompanied the administration of the provisions relating to sickness insurance; the machinery has on the whole worked much more smoothly and initial difficulties have been more easily overcome. An explanation of this probably lies in the fact that the administration is centralised, and a uniformity of practice thus secured which is impossible when benefits are administered by thousands of independent societies.

The first clause of the Bill effects a more or less minor amendment of the conditions attaching to the receipt of unemployment benefit. Under the principal Act the first of such conditions was that the insured person " proves that he has been employed as a workman in an insured trade in each of not less than twenty-six separate calendar weeks in the preceding five years." By virtue of the Amendment Bill it will in the future only be necessary to show that at least twenty contributions have been paid in respect of unemployment insurance, and these need not necessarily have been made during the preceding five years. A gain in simplicity is thus obtained, while the financial effect of the alteration will probably be insignificant. It should be borne in mind that although only twenty contributions are now required as against employment in each of twenty-six weeks under the original Act, yet the effect of casual or intermittent employment tends to make the conditions approximately financially the same. Employment for less than two days in a particular week, for instance, would formerly have had the effect of enabling the insured person to count that week as one of the necessary twenty-six, whereas now, the contribution not being a full one, *more* than twenty such weeks will be *required* to make up twenty contributions.

### Clauses of Interest to Employers.

The claim which probably concerns employers more than any other is Clause 5. This clause deals with the refund of contributions paid by an employer in respect of a workman continuously in his employment and simplifies the conditions attaching to such refund. The original Act allowed an employer (providing application was made within one month from the end of a calendar year) to claim from the unemployment fund a return of one-third of the contributions paid by him in respect of any of his workmen, provided that such workman had been *continuously in his service* throughout the preceding twelve months, and that not less than forty-five contributions had been paid in respect of him during that period. Apparently difficulty has arisen in connection with the practical application of this provision, and the main trouble appears to have been experienced with regard to the condition of *continuous* employment. Continuity of work without a break of any kind was obviously not intended (as the institution of a minimum number of contributions clearly shows), and the question thus arose, What causes of unemployment are to be regarded as not breaking continuity of service? Theoretically an answer is probably not difficult, but in practice innumerable unforeseen points arise.

Unemployment on account of illness, holidays, temporary slackness of work, or any other such cause, where, although not actually working, the employee may still be regarded as being employed by the same master, may fitly be considered as not disturbing continuity of employment. In order to obtain a refund, however, continuity of service must be *proved*, and as this proof carries with it the proof of causes of unemployment it can be seen that there might be considerable difficulty in substantiating a claim when application is only made yearly. To simplify matters, therefore, and also to avoid the trouble of calculating one-third of the total contributions paid by an employer in a year, it is now proposed by Clause 5 of the Amendment Bill that if an employer has paid, during any insurance year, forty-five contributions in respect of any of his workmen, he may claim from the unemployment fund three shillings in respect of each such workman, provided that application is made within two months of the close of such insurance year. The proviso as to continuous employment is thus entirely deleted, and the refund is made to depend only upon whether or not the stipulated minimum number of contributions have been paid. The substitution of " insurance year " for " calendar year " is, of course, an alteration made entirely in the interests of practical administration.

Another clause of the Bill in which employers will find much that concerns them is that which follows the one we have just discussed, and deals with the question of short-time employment. It is a common practice with large employers of labour in times of depression of trade to employ the whole or the large majority of their employees on short time instead of dismissing a proportion of them entirely from service. A certain amount of injustice would be caused in such cases to both employers and employed if contributions were required as in the case of full-time employment; it was, therefore, laid down in Section 96 of the principal Act that, subject to certain conditions, if an employer owing to slackness of trade keeps his workmen systematically employed on short time, and, in addition, pays in respect of them both his own and the employees' contributions, he would be entitled to claim from the unemployment fund the refund of the whole of the contributions so paid.

### The Method Examined.

The principle underlying this section is undoubtedly a good one, but there are obvious defects in the method adopted for carrying it out. The fact that an employer must first pay the contributions and then take steps to recover them is a cumbrous proceeding. Although the employer was entitled to obtain from the Board of Trade, beforehand, a ruling as to the circumstances under which the requirements of the section would be satisfied, it is obvious that the procedure laid down is somewhat clumsy, and has been found to be attended with administrative difficulties. The framers of the Amendment Bill have apparently recognised this, for Clause 6 of that Bill enables an employer to obtain from the Board of Trade an order, continuing in force for a stipulated period, exempting workmen employed by him, who are systematically working short time, from contributions under Part II. of the principal Act; the exemption applies to the contributions of both employer and employed. The payment and recovery of contributions and the incidental disadvantages attaching thereto are thus obviated.

The encouragement in this way afforded to employers to keep their workmen on short time rather than entirely to dismiss a proportion of them should be beneficial in its general effects, and the unemployment fund will lose nothing by the remission of contributions, since the payment of a certain amount of benefit is saved.

### The Same Principle and Sick Insurance.

It is open to question whether the same principle might not be extended still further, and contributions in respect of sickness insurance, or a part of them, be remitted in addition to those paid for unemployment insurance. Such remission could very well take place to the extent that the unemployment fund is saved the payment of benefit, though in this case it would probably be better from an administrative point of view for contributions to be first of all paid by the employer, and the amount then refunded to him out of the unemployment fund. No difficulty would then arise in connection with the crediting of contributions to the respective approved societies.

### Unemployment Benefit Granted by Associatons of Workmen.

Clauses 8 and 9 of the Bill introduce certain modifications with respect to the arrangements that may be made between the Board of Trade and Associations of workmen which pay unemployment benefit. Subject to certain conditions the Board of Trade are permitted to make arrangements with such Associations whereby the latter pay the same benefits as before the operation of the Act and reduce the subscriptions of such of their members as are "insured persons" by the amount of the statutory contributions, and the Board of Trade then pay in bulk to the Association the sum that would in the ordinary way have been payable direct to individual members. Clause 8 limits the Associations with which such arrangements can be made by stipulating that no arrangement can be made with an Association unless the unemployment benefit paid by such Association to members who are workmen in an insured trade, is, at least, one-third greater than the unemployment benefit provided by the Insurance Act.

### The Minor Changes.

The remaining clauses of the Bill institute, more or less, minor changes mainly of a technical nature; slight modifications are made with regard to the jurisdiction or procedure of the umpire and courts of referees; a clause is devoted to the procedure in proceedings for non-payment of contributions; the item "workmen" is further extended to include any person employed under a local or other public authority who would have been a workman within the meaning of that term as defined by the principal Act if the contract with the authority had been a contract of service; and a definition is given of persons who may act for the Board of Trade. The calculation of periods of benefit also receives attention.

For a concise description of the general effect of the Bill we cannot do better than quote the last paragraph of the Memorandum already referred to; it is there stated "that the net effect of the Bill is to afford a considerable measure of relief both to employers and to workmen's Associations, to simplify and thus cheapen administration, and to diminish rather than to increase the total charge upon the Treasury, while not prejudicing the financial position of the unemployment fund."

